# Effects of dietary PUFA patterns and FADS genotype on breast milk PUFAs in Chinese lactating mothers

**DOI:** 10.1186/s12263-023-00735-0

**Published:** 2023-10-25

**Authors:** Wen-Hui Xu, Yi-Ru Chen, Hui-Min Tian, Yi-Fei Chen, Jia-Yu Gong, Hai-Tao Yu, Guo-Liang Liu, Lin Xie

**Affiliations:** 1 Department of Nutrition and Food Hygiene, School of Public Health, Jilin University, No. 1163 Xinmin Street, Changchun, 130021 Jilin Province China; 2 Department of Clinical Nutrition, China-Japan, Union Hospital of Jilin University, Changchun, 130032 Jilin Province China; 3 School of Nursing, Jilin University, Changchun, 130021 Jilin Province China

**Keywords:** Dietary PUFA patterns, FADS polymorphism, Breast milk

## Abstract

**Background:**

Breastfeeding affects the growth and development of infants, and polyunsaturated fatty acids (PUFAs) play a crucial role in this process. To explore the factors influencing the PUFA concentration in breast milk, we conducted research on two aspects: dietary fatty acid patterns and single nucleotide polymorphisms (SNPs) in maternal fatty acid desaturase genes.

**Methods:**

Three hundred seventy Chinese Han lactating mothers were recruited. A dietary semi-quantitative food frequency questionnaire (FFQ) was used to investigate the dietary intake of lactating mothers from 22 to 25 days postpartum for 1 year. Meanwhile, breast milk samples were collected from the participants and tested for the concentrations of 8 PUFAs and 10 SNP genotypes. We sought to determine the effect of dietary PUFA patterns and SNPs on breast milk PUFAs. We used SPSS 24.0 statistical software for data analysis. Statistical tests were all bilateral tests, with *P* < 0.05 as statistically significant.

**Results:**

Under the same dietary background, PUFA contents in breast milk expressed by most major allele homozygote mothers tended to be higher than that expressed by their counterparts who carried minor allele genes. Moreover, under the same gene background, PUFA contents in breast milk expressed by the mother’s intake of essential PUFA pattern tended to be higher than that expressed by their counterparts who took the other two kinds of dietary.

**Conclusions:**

Our study suggests that different genotypes and dietary PUFA patterns affect PUFA levels in breast milk. We recommend that lactating mothers consume enough essential fatty acids to ensure that their infants ingest sufficient PUFAs.

**Supplementary Information:**

The online version contains supplementary material available at 10.1186/s12263-023-00735-0.

## Introduction

Breastfeeding affects infant growth and development [1]. For infants, exclusive breastfeeding is recommended for the first 6 months of life, with continued breastfeeding along with appropriate complementary food up to two years of age or beyond [2]. Breastfeeding is linked to positive health outcomes in both mothers and infants and favorable cognitive outcomes in infants [3].

Accumulating evidence indicates that polyunsaturated fatty acids (PUFAs) play a major role in brain development during the first year of life [4]. In vivo, the essential PUFA linoleic acid (C18:2n-6, LA) and α-linolenic acid (C18:3n-3, ALA) can be converted to n-6 and n-3 long-chain polyunsaturated fatty acids (LC-PUFAs) through elongation and desaturation reactions [5]. The dominant LC-PUFAs in these two families are docosahexaenoic acid (C22:6n-3, DHA) and arachidonic acid (C20:4n-6, AA). These fatty acids accumulate in the central nervous system [4] from the last trimester of pregnancy until the first year of life. Fatty acids of the n-3 family are essential for the development of the central nervous system, and DHA intake has been shown to be crucial for optimal visual development in formula-fed infants [6].

Breast milk is the only source of PUFAs for infants who are exclusively breastfed. The PUFA content depends on the intake of fatty acid precursors that cannot be produced in vivo [7]. PUFAs are metabolized by alternating desaturation and elongation reactions. These metabolic processes are limited by the enzymes 5-Δ-desaturase (D5D) and 6-Δ-desaturase (D6D), encoded by the fatty acid desaturase 1 (*FADS1*) and 2 (*FADS2*) genes. Several studies have demonstrated an association between the FADS enzymes and fatty acid levels [8–10]. The fluctuation of PUFA composition in breast milk may reflect metabolic adaptation in vivo to ensure a sufficient LC-PUFA supply to infants [11].

Examining dietary patterns has emerged as a holistic approach to capturing complex interactions between nutrients and foods [12]. One recent study on breast milk fatty acids considered the effect of single dietary fatty acids but overlooked the complex interactions between the many different fatty acids [13]. The dietary fatty acid pattern is the composition of multiple fatty acids [14] to evaluate the association between various fatty acid entries and then to identify potential hidden variables, namely dietary fatty acid patterns. However, dietary fatty acid patterns among lactating Chinese mothers remain largely underexplored. Therefore, this study aimed to explore the factors influencing breast milk PUFA content by analyzing the interaction between dietary fatty acid patterns and the single nucleotide polymorphisms (SNPs) of the maternal FADS gene. Thus, we hypothesized that the interaction between genes and dietary patterns would amplify the gap in knowledge about breast milk PUFA content.

## Materials and methods

### Research subject

All subjects of this study were healthy Chinese Han lactating mothers who received nursing care in a postpartum nursing center in Changchun from March 2012 to December 2014. The study was approved by the medical ethics committee of the School of Public Health of Jilin University, and all subjects signed informed consent. The inclusion criteria required that the nursing mother had no special eating habits, full-term delivery (37 ~ 42 weeks of pregnancy), infant Apgar score ≥ 8, and voluntary participation in this study. The exclusion criteria were pregnancy-induced hypertension, diabetes, and other metabolic diseases, including infectious diseases such as AIDS, tuberculosis, and other related drugs that affect the metabolism of nutrients. Babies suffering from breast-feeding contraindications were also excluded from the study. In total, 370 pregnant women were included as the research subjects.

### Questionnaire investigation

The sociodemographic information on the subjects was collected through questionnaires, including lactating mothers’ age, height, pre-pregnancy weight, postpartum weight, education level, and monthly household income. The type, quantity, and frequency of foods consumed by pregnant women in the past year were investigated by means of a semi-quantitative food frequency questionnaire (FFQ). In order to ensure the quality of the dietary survey, the special food pictures for the nutritional dietary survey, common food estimation tables, and quantitatively designed standard molds were used to verify the food intake, and the investigators were trained by a professional dietitian to conduct investigations and contact lactating mothers for clarification (if necessary). The questionnaire adopted a dual-entry method to avoid errors. The intake of dietary fatty acids was calculated according to the China Food Composition Tables (Sixth Edition) [15].

### Milk collection and detection of SNP typing of FADS gene cluster

Breast milk was collected between 8:00 a.m. and 11:00 a.m. on any day of the 22–25 days postpartum period. The entire milk from one breast was applied using an electric breast pump, and all samples were delivered to the laboratory by cryogenic transport and quickly transferred to a − 80℃ refrigerator for later testing. The detection of milk PUFAs is the same as the previous study of the research group [16]. The SNP genotyping method of the FADS gene cluster refers to the previous research of the research group [17]. SNPs in *FADS1*, *FADS2*, and *FADS3* were identified using the International HapMap Project SNP database and the National Center for Biotechnology Information (NCBI) (http://www.ncbi.nlm.nih.gov/snp/). Selected SNPs from the *FADS1* gene cluster (rs174547, rs174553), *FADS2* gene cluster (rs3834458, rs1535, rs174575, rs174602, rs498793) and *FADS3* gene cluster (rs174450, rs7115739, rs1000778) were genotyped using Sequenom Mass Array system (BO MIAO Biological Technological Company, Beijing) with validated primers. Each SNP had a minor allele frequency (MAF) above 10% in the Asian population according to the SNP database of the NCBI. The milk samples were thawed at 4℃, and genomic DNA was extracted from 300 μl of the cellular layer using the DNA kit (Beijing, TIANGEN) following the manufacturer’s instructions. The primer sequences of the ten SNPs in this study are shown in Supplementary Table 1.

### Statistical method

Data analyses were performed with SPSS 24.0 statistical software. Kolmogorov Smirnov test was used to judge whether the data meets the normal distribution. The measurement data meeting the normal distribution was represented by mean ± SD, and the measurement data of skewed distribution was represented by median (IQR); the count data was represented by component ratio. The principal component analysis (PCA) method was used to obtain the dietary fatty acid model. The rotation method used is the maximum variance method. The KMO test coefficient should be greater than 0.6 and the *P* value of Bartlett test should be less than 0.05. Chi-square test was used to compare the count data between groups. For the measurement data meeting the normal distribution, *t* test or ANOVA was used for the comparison between multiple groups, and the LSD method was used for the subsequent pairwise comparison; Kruskal–Wallis *H* test was used for multi-group comparison of skewed distribution measurement data, and Bonferroni method was used for subsequent pairwise comparison. Statistical tests were all bilateral tests, with *P* < 0.05 as the difference, which was statistically significant.

## Result

### Subject characteristics

A total of 370 healthy lactating mothers aged 30 years participated in this study. Their monthly family income was divided into 2000 ~ 5000 yuan, 5000 ~ 10,000 yuan, and above 10,000 yuan, accounting for 15.5%, 53.5%, and 31.0%, respectively. Regarding education, 43.8% of the participants had a high school education or below, 46.3% had a bachelor’s degree, and 9.9% had a graduate degree or above. Lactating mothers with normal BMI before pregnancy accounted for 61.3%, gaining 18.5 kg during pregnancy and 39.1 weeks of gestation. The birth weight of the infant was 3.4 kg, and their body length was 50.0 cm. Details are shown in Table 1.

**Table 1:** Demographic and clinical characteristics of mothers and infants (*N* = 370)

Variable	Median (IQR)/*N* (%)
Mother	
Age (year)^a^	30.0 (4.0)
Educational level	
Postgraduate and above	35 (9.9)
Bachelor’s degree	163 (46.3)
High school and below	154 (43.8)
Monthly household income (yuan)	
2000 ~	46 (15.5)
5000 ~	159 (53.5)
10,000 ~	92 (31.0)
Pre-pregnancy weight (kg)^a^	55.0 (10.0)
Pre-pregnancy BMI (kg/m^2^)	
< 18.5	85 (23.5)
18.5 ~ 23.9	222 (61.3)
24 ~ 27.9	42 (11.6)
≥ 28	13 (3.6)
Weight gain during pregnancy (kg)^a^	18.5 (7.0)
Gestational week (week)^a^	39.1 (1.1)
Infant	
Gender	
Male	186 (50.8)
Female	180 (49.2)
Weight (kg)^a^	3.4 (0.5)
Length (cm)^a^	50.0 (1.0)

*IQR* inter-quartile range, *BMI* body mass index

^a^Values are median (IQR) for skewed distributed variables

### Dietary fatty acid intake

Supplementary Table 2 showed the comparison between the dietary PUFA intake of the subjects obtained from the dietary frequency questionnaire and their adequate intake (AI), in which the average intake of LA was 18.88 g/d and the average intake of ALA was 1.78 g/d, which was higher than the AI, accounting for 185.10% and 116.34% of the AI separately; the average intake of dietary eicosapentaenoic acid (C20:5n-3, EPA) was 10.82 mg/d, accounting for 21.64% of the AI, and the average intake of dietary DHA was 15.54 mg/d, which was lower than the recommended AI, accounting for only 7.8% of the AI.

### Dietary fatty acid pattern

In this study, the dietary fatty acid patterns of 370 subjects were analyzed by PCA. There was a strong linear correlation between the research variables and the data structure was reasonable (KMO test coefficient was 0.69, Bartlett test result was *χ*^2^ = 10,414.pc098, *P* < 0.001), suggesting that the PCA method can be used.

The first three principal component eigenvalues in this study were greater than 1, explaining 45.25%, 18.89%, and 12.23% of the total data variance, respectively, and accurately reflected the dietary fatty acid patterns of lactating mothers. After extraction, the principal components accounted for 76.37% of the total data variation. The rotating component matrix of the dietary fatty acid model outputs the interpretation of the variables for each principal component after extraction. The first principal component mainly included C22:6n-3, C18:4n-3, C20:5n-3, C22:5n-3, and C20:4n-6; therefore, the model dominated by n-3 PUFAs was defined as the n-3 PUFA major pattern. The second main component involved C22:4n-6, C22:3n-3, and C20:3n-6, which was defined as the n-6 PUFAs major pattern. The third principal component mainly involved LA and ALA; therefore, the model dominated by essential PUFAs was defined as the essential PUFA pattern. For each participant, three values for the three different patterns were calculated, and the maximum value represented the dietary fatty acid pattern (Table 2).

**Table 2:** Rotating component matrix of dietary fatty acid pattern

Dietary fatty acids	n-3 PUFA major pattern (*N* = 128)	n-6 PUFA major pattern (*N* = 82)	Essential PUFA pattern (*N* = 160)
C22:6n-3 (DHA)	**0.999**	0.020	-0.010
C18:4n-3 (SDA)	**0.999**	- 0.002	-0.012
C20:5n-3 (EPA)	**0.998**	0.035	0.011
C22:5n-3 (DPA)	**0.998**	0.040	-0.009
C20:4n-6 (AA)	**0.985**	0.124	0.011
C22:4n-6	0.018	**0.940**	-0.005
C22:3n-3 (DTA)	-0.013	**0.937**	0.013
C20:3n-6 (DGLA)	0.052	**0.445**	0.154
C18:3n-3 (ALA)	0.065	0.119	**0.831**
C18:2n-6 (LA)	0.032	0.228	**0.753**
C20:2n-6 (EDA)	-0.095	-0.182	0.260

*PUFA* polyunsaturated fatty acids, *C22:6n-3* docosahexaenoic acid, DHA, *C18:4n-3* stearidonic acid, SDA, *C20:5n-3* Eicosapentaenoic acid, EPA, *C22:5n-3* docosapentaenoic acid, DPA, *C20:4n-6* Arachidonic acid, AA, *C22:4n-6* docosatetraenoic acid, *C22:3n-3* docosatrienoic acid, DTA, *C20:3n-6* dihomo-γ-linolenic acid, DGLA, *C18:3n-3* α-linolenic acid, ALA, *C18:2n-6* linoleic acid, LA, *C20:2n-6* eicosadienoic acid, EDA

### PUFAs in breast milk

The breast milk fatty acid content of the 370 lactating mothers is shown in Table 3. Average breast milk LA content was 0.378 ± 0.192 (g/100g), average breast milk γ-linoleic acid (GLA) content was 0.038 ± 0.032 (g/100g), average breast milk dihomo-γ-linolenic acid (DGLA) was 0.052 ± 0.040 (g/100g), average breast milk AA was 0.081 ± 0.041 (g/100g), average breast milk DTA was 0.019 ± 0.010 (g/100g), average breast milk ALA was 0.146 ± 0.076 (g/100g), average breast milk EPA was 0.008 ± 0.007 (g/100g), and average breast milk DHA was 0.049 ± 0.035 (g/100g).

**Table 3:** The levels of PUFA in breast milk of 370 lactating mothers (g/100g)

Fatty acids	Mean/median	SD/IQR
LA^a^	0.378	0.192
GLA^b^	0.038	0.032
DGLA^b^	0.052	0.040
AA^a^	0.081	0.041
DTA^b^	0.019	0.010
ALA^a^	0.146	0.076
EPA^b^	0.008	0.007
DHA^b^	0.049	0.035
n-6 PUFAs	0.575	0.279
n-3 PUFAs	0.209	0.107
n-6/n-3	2.834	0.517

*PUFAs* polyunsaturated fatty acids, *IQR* inter-quartile range, *LA* linoleic acid, *GLA* γ-linoleic acid, *DGLA* dihomo-γ-linolenic acid, *AA* arachidonic acid, *DTA* docosatetraenoic acid, *ALA* α-linolenic acid, *EPA* eicosapentaenoic acid, *DHA* docosahexenoic acid

^a^Values are mean ± SD for normal distribution

^b^Values are median (IQR) for skewed distributed variables

### Characteristics of SNP

Ten SNPs were identified in the present study. Table 4 lists the specific characteristics of the ten candidate SNPs in the FADS gene cluster. The distributions of genotype frequencies in the 422 subjects were in Hardy–Weinberg equilibrium (*P* > 0.05).

**Table 4:** Characteristics of the SNPs

SNP	Position	Allele M/m	Genotype (*n*)			MAF (%)	Genotyping success rate (%)
			M/M	M/m	m/m		
rs174547	61,803,311	G/T	172	148	35	30.70	95.95
rs174553	61,807,686	T/C	176	152	35	30.58	98.11
rs3834458	61,827,449	T/del	174	150	37	31.02	97.57
rs1535	61,830,500	A/G	170	158	38	31.97	98.92
rs174575	61,834,531	C/G	300	59	5	9.48	93.38
rs174602	61,856,942	T/C	218	125	19	22.51	97.84
rs498793	61,857,233	C/T	310	50	6	8.47	98.92
rs174450	61,874,070	A/G	206	122	36	26.65	98.38
rs7115739	61,874,245	G/T	254	88	20	17.68	97.84
rs1000778	61,887,833	G/A	232	111	20	20.80	98.11

*SNP* single-nucleotide polymorphism, *MAF* minor allele frequency, *M/M* major/major allele, *M/m* major/minor allele, *m/m* minor/minor allele, *del* deletion

**Fig. 1:**
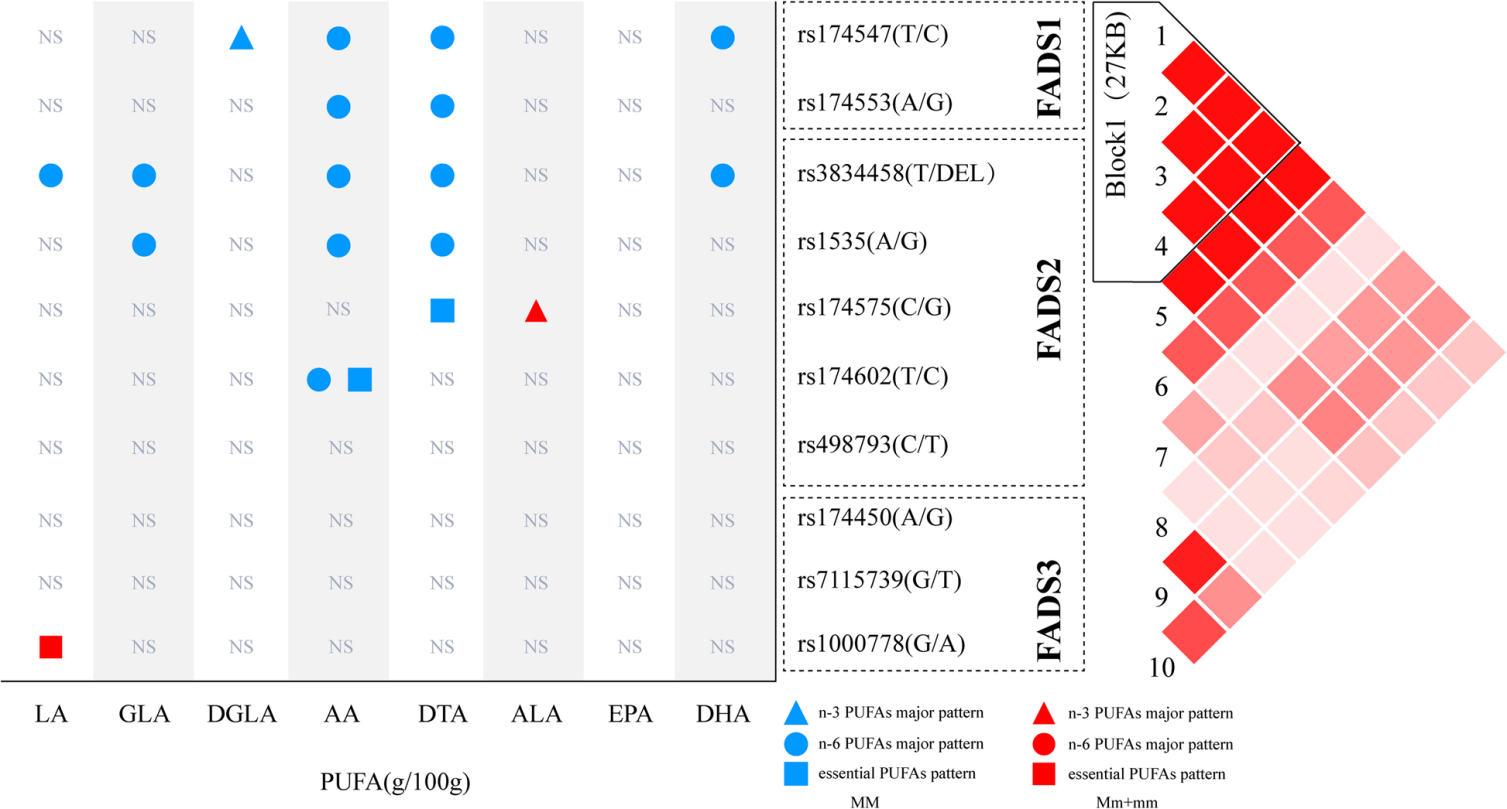
Effects of the same dietary PUFA pattern and different genotypes on breast milk PUFAs. The blue color indicates that the content of this type of PUFA in breast milk was higher in MM than in Mm/mm (MM > Mm/mm). Red indicates that the content of this type of fatty acid in breast milk was lower in MM than in Mm/mm (MM < Mm/mm). NS indicates that no significant differences were present in the three dietary patterns in breast milk PUFAs between MM and Mm/mm under the same dietary PUFA pattern (*P* > 0.05). Triangles represent the n-3 PUFA major pattern, circles represent the n-6 PUFA major pattern, and squares represent the essential PUFA pattern. In the figure, only the symbols of the models with statistical differences (*P* < 0.05) are shown; the symbols of the models without statistical differences (*P* > 0.05) are not shown

As shown in Fig. 1, in the n-3 PUFAs major pattern, the concentration of DGLA in breast milk was higher in major allele homozygotes (MM) than in minor allele carriers (Mm/mm) for the *FADS1* rs174547 gene (*P* = 0.019), whereas the concentration of ALA (*P* = 0.006) in breast milk was lower in MM than in Mm/mm for the *FADS2* rs174575 gene. In the n-6 PUFAs major pattern, the concentration of AA (*P* = 0.003), DTA (*P* = 0.019), and DHA (*P* = 0.032) in breast milk was higher in MM than in Mm/mm with the *FADS1* rs174547 gene; the concentration of AA (*P* = 0.005) and DTA (*P* = 0.026) in breast milk was higher in MM than in Mm/mm with the *FADS1* rs174553 gene; the concentration of LA (*P* = 0.040), GLA (*P* = 0.042), AA (*P* = 0.002), DTA (*P* = 0.009), and DHA (*P* = 0.021) in breast milk was higher in MM than in Mm/mm with the *FADS2* rs3834458 gene; the concentration of GLA (*P* = 0.047), AA (*P* = 0.004), and DTA (*P* = 0.022) in breast milk was higher in MM than in Mm/mm with the *FADS2* rs1535 gene; and the concentration of AA (*P* = 0.045) in breast milk was higher in MM than in Mm/mm with the *FADS2* rs174602 gene. In the essential PUFAs pattern, the concentration of AA in breast milk for the *FADS2* rs174602 (*P* = 0.040) gene was higher in MM than in Mm/mm, and DTA was higher in MM than in Mm/mm for the *FADS2* rs174575 gene (*P* = 0.023), but the concentration of LA in breast milk was lower in MM than in Mm/mm for the *FADS3* rs1000778 (*P* = 0.023) gene. In addition, within the same dietary pattern, the concentration of PUFAs in breast milk did not differ between MM and Mm/mm of *FADS2* rs498793, *FADS3* rs174450, and *FADS3* rs7115739.

**Fig. 2:**
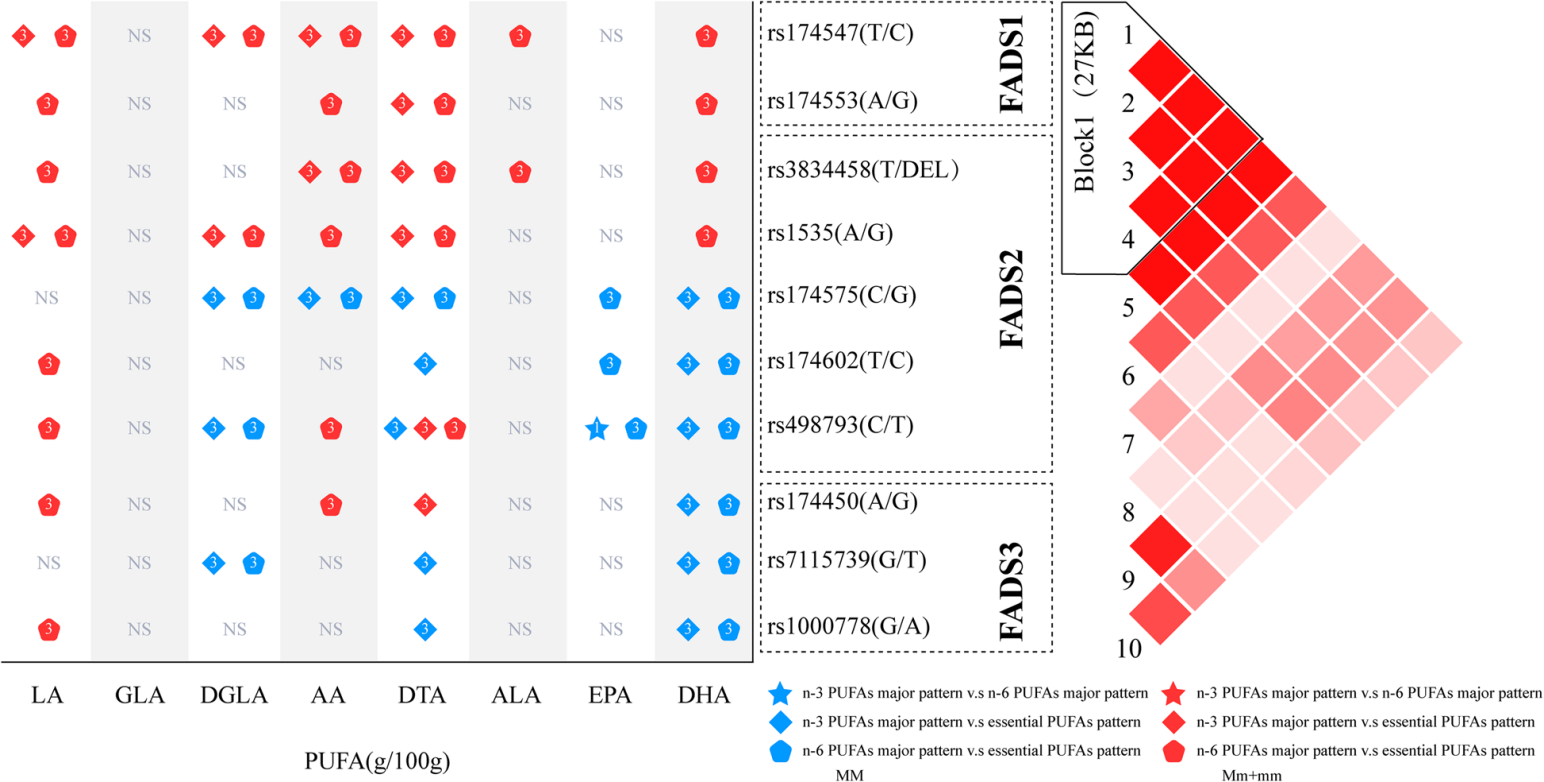
Effects of different dietary patterns of the same genotype on breast milk PUFAs. Blue indicates the major allele homozygotes, while red indicates minor allele carriers. NS indicates that no significant differences were present in the PUFAs in breast milk of lactating mothers with different dietary patterns under the same two genotypes. The star indicates that dietary model 1 was compared with the n-6 PUFAs major pattern, the diamond indicates that the n-3 PUFA major pattern was compared with the essential PUFAs pattern; the pentagon indicates that the n-6 PUFAs major pattern was compared with the essential PUFAs pattern; and the number in the symbol indicates the dietary model with higher breast milk PUFAs. In the figure, only the symbols of the models with statistical differences are shown; the symbols of the models without statistical differences are not shown

Overall, as shown in Fig. 2, mothers carrying the minor alleles (Mm/mm) of the *FADS1* rs174547, *FADS1* rs174553, *FADS2* rs3834458, and *FADS2* rs1535 genes had significantly higher concentrations of LA, DGLA, AA, DTA, ALA, and DHA in their breast milk than mothers who were homozygous for the major allele (MM). Further analysis showed that the levels of PUFAs in the breast milk of mothers carrying the minor allele (Mm/mm) did not differ significantly across dietary patterns. In addition, regardless of the lactating mother’s adoption of any of the three dietary patterns, compared with Mm/mm, the milk of mothers homozygous for the major allele (MM) of the *FADS2* rs174575 and *FADS3* rs7115739 genes was significantly different for DGLA, AA, DTA, EPA, and DHA, which were significantly higher in the breast milk of their counterparts. Similarly, the concentrations of PUFAs in the breast milk of mothers homozygous for the major allele (MM) did not differ significantly across dietary patterns.

For mothers carrying the *FADS2* rs174602 and *FADS3* rs1000778 minor alleles (Mm/mm) and ingesting the essential PUFAs pattern, the concentration of LA in the breast milk was significantly higher than that in mothers carrying the same genotype and ingesting the other two dietary patterns. However, the concentrations of DTA, EPA, and DHA in breast milk with the essential PUFA pattern were the highest among the three patterns homozygous for the major allele (MM). Mothers who consumed the essential PUFAs pattern and were homozygous for the *FADS2* rs498793 major allele (MM) had significantly higher concentrations of DGLA and DHA in their breast milk than mothers of the same genotype who chose the other two dietary patterns. Mothers who consumed the essential PUFA pattern and carried the *FADS2* rs498793 minor allele (Mm/mm) had significantly higher concentrations of DTA in their breast milk than mothers of the same genotype who chose the other two dietary patterns. In addition, mothers who consumed the n-3 PUFAs dietary pattern and carried the *FADS2* rs498793 allele had significantly lower EPA concentrations in their breast milk than mothers with the same genotype who chose the n-6 PUFAs dietary pattern. Among the Mm/mm of the three patterns, mothers who consumed the essential PUFA pattern and carried the *FADS3* rs174450 minor allele (Mm/mm) had significantly higher concentrations of LA, AA, and DTA in their breast milk than those who consumed the n-6 PUFA major pattern with the same genotype; in contrast, mothers who selected the essential PUFA pattern carrying the MM had significantly higher concentrations of DHA in their breast milk than those who consumed the other two dietary patterns with the same genotype. No differences were observed in other PUFAs in the breast milk of mothers homozygous for both MM and Mm/mm under different dietary patterns. The details are shown in Fig. 2. (The specific values are described in detail in Supplementary Table 3– 12).

## Discussion

The World Health Organization recommends that mothers worldwide breastfeed their babies during the first 6 months of life for optimal growth, development, and health [18]. PUFAs are important nutritional substances that regulate brain development, and PUFA insufficiency may impair cognition and disease including cardiovascular disease, cancer, and diabetes [19–22]. Billeaud et al. reported that the intake of LA, ALA, and LA/ALA can affect PUFA composition in breast milk. Additionally, the genotype also affects breast milk PUFA content [23–27]. Studies on the blood of pregnant women in India have found that dietary patterns regulate the expression of *FADS1* [28]; however, few studies have concentrated on breast milk fatty acids affected by both dietary patterns and genotypes.

This study found that in the same dietary model, the concentration of PUFAs in breast milk of *FADS1* rs174547, *FADS1* rs174553, *FADS2* rs3834458, *FADS2* rs1535, and *FADS2* rs174602 MM was significantly higher than that of Mm/mm, and the content of DTA in breast milk of *FADS2* rs174575 MM was significantly higher than that of Mm/mm, indicating that the genotype would affect the content of fatty acids in breast milk. Compared with MM, the concentration of PUFAs secreted in the breast milk of Mm/mm was less; therefore, additional supplementation is needed for infants to have enough fatty acids to ensure adequate nutrition. Studies have found that the proportion of DHA in the breast milk of Mm/mm of *FADS1* rs174561, *FADS2* rs174575, and *FADS2* rs3834458 is lower than that in MM, which are findings similar to our study [8]. The content of some PUFAs in breast milk showed that the MM was significantly higher than the Mm/mm in the *FADS1* rs174547, *FADS1* rs174553, *FADS2* rs3834458, and *FADS2* rs1535 genotypes, which was consistent with linkage disequilibrium between them.

The ALA content in the breast milk of *FADS2* rs174575 MM was significantly lower than that of Mm/mm, and the concentration of LA in the breast milk of *FADS3* rs1000778 MM was significantly lower than that of Mm/mm, indicating that the genotype might affect the concentration of PUFAs in breast milk. ALA and LA are precursors of other PUFAs; therefore, if a mother is a Mm/mm, the activity of the desaturase enzyme is weak and the amount of other PUFAs is low; therefore, the content of ALA and LA in breast milk is abundant. Compared with MM, the content of PUFAs secreted by lactating mothers of Mm/mm was adequate. O’Neill’s et al. reported similar results for plasma [29]. No significant differences in the fatty acid content of breast milk between MM and Mm/mm for *FADS2* rs498793, *FADS3* rs174450, and *FADS3* rs7115739 were observed, indicating that these three SNPs did not affect the concentration of PUFAs in breast milk. In general, lactating mothers of different genotypes need personalized and accurate nutrition to ensure that they secrete milk with a more balanced nutrition to ensure the healthy growth of infants.

This study found no significant differences in breast milk fatty acids among *FADS1* rs174547, *FADS1* rs174553, *FADS2* rs1535, and *FADS2* rs383445 MM in the three dietary patterns, but differences in breast milk fatty acids were found among Mm/mm in the three dietary patterns, which further explained that the genotype would affect the content of breast milk fatty acids and that the MM would not be affected by the diet; therefore, ample PUFAs in their breast milk would be present. However, some PUFAs in the breast milk produced by Mm/mm were insufficient, indicating that it is necessary to adjust the diet for additional supplements to ensure that infants have sufficient fatty acid intake. No significant differences in breast milk fatty acids were observed between *FADS2* rs174575 and *FADS3* rs7115739 Mm/mm in the three dietary patterns; however, differences in breast milk fatty acids between MM in the three dietary patterns, which showed that the genotype affected the content of breast milk fatty acids. Mm/mm was not affected by diet and had adequate PUFAs in their breast milk.

Mothers who were MM of *FADS2* rs174602, *FADS2* rs498793, *FADS3* rs1000778, and *FADS3* rs174450 had significantly higher PUFA levels in their breast milk than the other two dietary patterns when consuming an essential PUFA dietary pattern. Therefore, we inferred that the precursor ratio in the diet affected the PUFAs in the breast milk of MM. Among different dietary patterns of the same genotype, fatty acids in most breast milk samples were higher in the essential PUFA pattern. A possible reason is that the essential PUFA pattern is dominated by the essential fatty acids ALA and LA. When the lactating mother is a MM, her desaturase activity is strong, resulting in a higher content of PUFAs such as AA in breast milk. When the lactating mother is a Mm/mm, her desaturase activity is weak, resulting in a higher content of the substrate itself (LA and ALA).

The limitations of this study are as follows: (1) only eight PUFAs were detected in breast milk, and 11 PUFAs were used for dietary pattern classification, which is not comprehensive; (2) The PUFAs used in this study for dietary pattern classification did not completely match those detected in breast milk. (3) We conducted a dietary pattern survey using the FFQ without conducting a 3-day and 24-h questionnaire simultaneously, which cannot accurately control the intake of PUFAs in lactating mothers, as in a randomized controlled trial [24]; (4) The research objectives were assigned to each group. The number of individual groups was small and the number of patients in each group was not uniform. The sample size should be expanded further to ensure the accuracy and precision of the results.

The main advantages of this study are as follows: (1) Previous studies have primarily focused on the effects of different diets [30–33] or genotypes [33–36] on breast milk fatty acids. Our study combined diets and genotypes to explore their comprehensive effects on breast milk fatty acids, reflecting a real-world phenomenon; (2) Previous studies analyzed dietary fatty acids separately from those in breast milk [31]. This study first classified dietary fatty acids and then analyzed fatty acids in breast milk, which is more in line with the actual situation and makes it easier to find regular changes, facilitating the presentation of valuable information to direct future research and clinical applications.

## Conclusion

Our study suggests that different genotypes and dietary PUFA patterns affect PUFA levels in breast milk. In addition, desaturase activity in the major allele homozygotes is stronger, and more downstream products are metabolized in breast milk, especially in mothers who consume essential fatty acid dietary patterns. Therefore, we recommend that lactating mothers consume enough essential fatty acids to ensure that their infants ingest sufficient PUFAs. In the future, we will conduct a randomized study with greater precision of dietary intake by varying the known quantities of PUFAs in groups of mothers with different genetic variants.

### Supplementary Information

**Additional file 1: Supplementary Table S1.** The primer sequences of ten SNPs in *FADS* genes (5'-3'). **Supplementary Table 2.** Dietary polyunsaturated fatty acid intake of lactating mothers. **Supplementary Table 3.** Effects of rs174547 dominant pattern of *FADS1* gene and different dietary patterns on fatty acid concentration in breast. **Supplementary Table 4**. Effects of rs174553 dominant pattern of *FADS1* gene and different dietary patterns on fatty acid concentration in breast. **Supplementary Table 5.** Effects of rs1535 dominant pattern of *FADS2* gene and different dietary patterns on fatty acid concentration in breast milk. **Supplementary Table 6.** Effects of rs174575 dominant pattern of *FADS2* gene and different dietary patterns on fatty acid concentration in breast milk. **Supplementary Table 7.** Effects of rs174602 dominant pattern of *FADS2* gene and different dietary patterns on fatty acid concentration in breast. **Supplementary Table 8.** Effects of rs3834458 dominant pattern of *FADS2* gene and different dietary patterns on fatty acid concentration in breast milk. **Supplementary Table 9.** Effects of rs498793 dominant pattern of *FADS2* gene and different dietary patterns on fatty acid concentration in breast milk. **Supplementary Table 10.** Effects of rs1000778 dominant pattern of *FADS3* gene and different dietary patterns on fatty acid concentration in breast milk. **Supplementary Table 11.** Effects of rs174450 dominant pattern of *FADS3* gene and different dietary patterns on fatty acid concentration in breast milk. **Supplementary Table 12.** Effects of rs7115739 dominant pattern of *FADS3* gene and different dietary patterns on fatty acid concentration in breast milk.

## Data Availability

All data generated or analyzed during this study are included in this published article [and its supplementary information files].
