# Supplementary material for: Effects of dietary PUFA patterns and FADS genotype on breast milk PUFAs in Chinese lactating mothers
Source: Genes Nutr. 2023 Oct 25;18:16. doi: 10.1186/s12263-023-00735-0 (PMC10598896; doi:10.1186/s12263-023-00735-0)
Supplement: Supplementary file 1 — Additional file 1: Supplementary Table S1. The primer sequences of ten SNPs in FADS genes (5'-3'). Supplementary Table 2. Dietary polyunsaturated fatty acid intake of lactating mothers. Supplementary Table 3. Effects of rs174547 dominant pattern of FADS1 gene and different dietary patterns on fatty acid concentration in breast. Supplementary Table 4. Effects of rs174553 dominant pattern of FADS1 gene and different dietary patterns on fatty acid concentration in breast. Supplementary Table 5. Effects of rs1535 dominant pattern of FADS2 gene and different dietary patterns on fatty acid concentration in breast milk. Supplementary Table 6. Effects of rs174575 dominant pattern of FADS2 gene and different dietary patterns on fatty acid concentration in breast milk. Supplementary Table 7. Effects of rs174602 dominant pattern of FADS2 gene and different dietary patterns on fatty acid concentration in breast. Supplementary Table 8. Effects of rs3834458 dominant pattern of FADS2 gene and different dietary patterns on fatty acid concentration in breast milk. Supplementary Table 9. Effects of rs498793 dominant pattern of FADS2 gene and different dietary patterns on fatty acid concentration in breast milk. Supplementary Table 10. Effects of rs1000778 dominant pattern of FADS3 gene and different dietary patterns on fatty acid concentration in breast milk. Supplementary Table 11. Effects of rs174450 dominant pattern of FADS3 gene and different dietary patterns on fatty acid concentration in breast milk. Supplementary Table 12. Effects of rs7115739 dominant pattern of FADS3 gene and different dietary patterns on fatty acid concentration in breast milk. [file 12263_2023_735_MOESM1_ESM.docx]

**Supplementary Table 1 The primer sequences of ten SNPs in *FADS* genes (5'-3')**

| SNP |  | Sequence |
| --- | --- | --- |
| rs 174547 | Forward | ACGTTGGATGAGACTGGAGCATAACACAAC |
|  | Reverse | ACGTTGGATGGAGCCTCAGGCTAATGAGAA |
|  | UEP | CTGTTTTCACCTACGCA |
| rs 174553 | Forward | ACGTTGGATGATGGAGTGAGACAGCAGAAC |
|  | Reverse | ACGTTGGATGACCATGTCAACCAGCCAGTC |
|  | UEP | GTGGCCAGTCTAGAACCCCTG |
| rs 174602 | Forward | ACGTTGGATGAGCAGATAGAAGGGATGGTG |
|  | Reverse | ACGTTGGATGAGATCATGAGCACCCAGTAG |
|  | UEP | CATGTTCCCCAACCC |
| rs 3834458 | Forward | ACGTTGGATGCCTTGGATTAGAGGGCTTTG |
|  | Reverse | ACGTTGGATGACCAAGAAAGCAGAGCAGAG |
|  | UEP | TAAGAGGTTCCGCAATTCTTTTC |
| rs 1000778 | Forward | ACGTTGGATGGGTCTGGGATTTGAATCCAC |
|  | Reverse | ACGTTGGATGGAGTGCCGGAAATAAACAGG |
|  | UEP | GCAGCATAGCATGCCCTT |
| rs 498793 | Forward | ACGTTGGATGACTCTCTGAGCCTCTGTGTC |
|  | Reverse | ACGTTGGATGCTAAACTTGTTAGAAGCGGG |
|  | UEP | CCCATCAGGCCTGTAAC |
| rs 174575 | Forward | ACGTTGGATGTTCCAACTCCAAGGGAGCAG |
|  | Reverse | ACGTTGGATGCGAGAGCCCTCTGGAAATG |
|  | UEP | CTGCCTTGACCTTGGTTGCT |
| rs 1535 | Forward | ACGTTGGATGCCCTCCAAAAATCAGTGCTC |
|  | Reverse | ACGTTGGATGGAGACAGAGAAAAGGTCAGG |
|  | UEP | TCTCCTAACAGAGGACTAG |
| rs 174450 | Forward | ACGTTGGATGGCTGAATGCTCATTCTCTGG |
|  | Reverse | ACGTTGGATGATGCCTGGACTTGGCCTTG |
|  | UEP | TGGAATCTAGACCGGG |
| rs 7115739 | Forward | ACGTTGGATGAACCCAAGGACAGAGAGGA |
|  | Reverse | ACGTTGGATGGGCTTCCTAGCAGATTGTTG |
|  | UEP | GGGCTCTTGGGCCTGATATTGG |

SNP, Single nucleotide polymorphism.

**Supplementary Table 2 Dietary polyunsaturated fatty acid intake of lactating mothers**

| Dietary fatty acids | Dietary intake  Median (IQR) | AI ^a^ | Adequacy ^c^ (%) |
| --- | --- | --- | --- |
| C18:2n-6 (g) | 18.88 (8.50) | 10.20 ^b^ | 185.10 |
| C18:3n-3 (g) | 1.78 (1.27) | 1.53 ^b^ | 116.34 |
| C18:4n-3 (g) | 0.00 (0.00) | - | - |
| C20:2n-6 (mg) | 119.25 (103.01) | - | - |
| C20:3n-6 (mg) | 0.38 (1.03) | - | - |
| C20:4n-6 (mg) | 10.73 (19.62) | - | - |
| C20:5n-3 (mg) | 10.82 (25.61) | 50 | 21.64 |
| C22:3n-3 (mg) | 0.09 (0.51) | - | - |
| C22:4n-6 (mg) | 0.84 (3.60) | - | - |
| C22:5n-3 (mg) | 1.54 (4.15) | - | - |
| C22:6n-3 (mg) | 15.54 (36.23) | 200 | 7.80 |

IQR, Inter-quartile range; C18:2n-6, Linoleic acid, LA; C18:3n-3, α-linolenic acid, ALA; C18:4n-3, Stearidonic acid, SDA; C20:2n-6, Eicosadienoic acid, EDA; C20:3n-6, Dihomo-γ-linolenic acid, DGLA; C20:4n-6, Arachidonic acid, AA; C20:5n-3, Eicosapentaenoic acid, EPA; C22:3n-3, Docosatrienoic acid, DTA; C22:4n-6, Docosatetraenoic acid; C22:5n-3, Docosapentaenoic acid, DPA; C22:6n-3, Docosahexaenoic acid, DHA.

^a^ AI: adequate intake, According to *Dietary reference intakes for China (2023 Edition)*.

^b^ The recommended dietary amounts of C18:2n-6 and C18:3n-3 are 4.0% and 0.6% of the total energy intake respectively, and each gram of fat can provide 9kcal of energy.

^c^ Adequacy: (average dietary intake / recommended dietary intake)×100%.

**Supplementary Table 3 Effects of rs174547 dominant pattern of *FADS1* gene and different dietary patterns on fatty acid concentration in breast milk**

| *FADS1*  rs174547 | LA | GLA | ALA | DGLA | AA | EPA | DTA | DHA |
| --- | --- | --- | --- | --- | --- | --- | --- | --- |
| TT+M1 | 0.387±0.206 | 0.044  (0.026,0.066) | 0.141±0.073 | 0.057±0.030 | 0.080  (0.053,0.116) | 0.009  (0.005,0.013) | 0.018±0.010 | 0.051±0.028 |
| TCCC+M1 | 0.353±0.191 | 0.037  (0.022,0.053) | 0.146±0.086 | 0.045±0.025^a^ | 0.068  (0.044,0.097) | 0.008  (0.005,0.010) | 0.016±0.008 | 0.045±0.021 |
| TT+M2 | 0.389±0.219 | 0.038  (0.026,0.053) | 0.151±0.088 | 0.053  (0.030,0.077) | 0.092±0.053 | 0.007  (0.003,0.012) | 0.021  (0.011,0.028) | 0.054±0.031 |
| TCCC+M2 | 0.312±0.136 | 0.030  (0.018,0.048) | 0.125±0.062 | 0.040  (0.026,0.062) | 0.061±0.028^a^ | 0.006  (0.004,0.010) | 0.014  (0.010,0.021)^a^ | 0.040±0.020^a^ |
| TT+M3 | 0.392±0.186 | 0.042  (0.029,0.064) | 0.151±0.075 | 0.057  (0.040,0.081) | 0.092±0.040 | 0.009  (0.006,0.013) | 0.021  (0.014,0.029) | 0.057  (0.036,0.071) |
| TCCC+M3 | 0.414±0.181^bde^ | 0.039  (0.025,0.048) | 0.160±0.071^be^ | 0.054  (0.037,0.069)^bde^ | 0.084±0.038^bde^ | 0.007  (0.005,0.011) | 0.018  (0.014,0.027)^bde^ | 0.053  (0.035,0.075)^be^ |

^a^ indicates that there is a statistical difference in the content of breast milk PUFAs in different genotypes of the same dietary model.

^b^ indicates that there is a statistical difference in breast milk PUFAs in different dietary models of the same genotype.

^c^ indicates that there is a statistical difference in breast milk PUFAs between model 1 and model 2 with the same genotype.

^d^ indicates that there is a statistical difference in breast milk PUFAs between model 1 and model 3 with the same genotype.

^e^ indicates that there is a statistical difference in breast milk PUFAs between model 2 and model 3 with the same genotype.

**Supplementary Table 4 Effects of rs174553 dominant pattern of *FADS1* gene and different dietary patterns on fatty acid concentration in breast milk**

| *FADS1*  rs174553 | LA | GLA | ALA | DGLA | AA | EPA | DTA | DHA |
| --- | --- | --- | --- | --- | --- | --- | --- | --- |
| AA+M1 | 0.364±0.211 | 0.042  (0.020,0.062) | 0.132±0.074 | 0.054±0.031 | 0.078  (0.043,0.109) | 0.008  (0.004,0.013) | 0.017±0.011 | 0.048±0.028 |
| AGGG+M1 | 0.364±0.190 | 0.038  (0.023,0.052) | 0.151±0.088 | 0.047±0.026 | 0.070  (0.046,0.098) | 0.008  (0.005,0.010) | 0.017±0.008 | 0.046±0.021 |
| AA+M2 | 0.389±0.223 | 0.042±0.025 | 0.151±0.089 | 0.053  (0.030,0.078) | 0.092±0.054 | 0.007  (0.003,0.012) | 0.021  (0.010,0.028) | 0.053±0.031 |
| AGGG+M2 | 0.316±0.135 | 0.033±0.016 | 0.127±0.062 | 0.041  (0.030,0.078) | 0.062±0.028^a^ | 0.006  (0.004,0.010) | 0.014  (0.010,0.021)^a^ | 0.041±0.020 |
| AA+M3 | 0.394±0.188 | 0.043  (0.029,0.064) | 0.151±0.075 | 0.058  (0.040,0.081) | 0.092±0.041 | 0.009  (0.006,0.013) | 0.021±0.009 | 0.057  (0.036,0.072) |
| AGGG+M3 | 0.422±0.191^be^ | 0.039  (0.027,0.049) | 0.157±0.066 | 0.055  (0.037,0.070) | 0.084±0.038^be^ | 0.007  (0.005,0.012) | 0.020±0.010^bde^ | 0.051  (0.035,0.069)^be^ |

^a^ indicates that there is a statistical difference in the content of breast milk PUFAs in different genotypes of the same dietary model.

^b^ indicates that there is a statistical difference in breast milk PUFAs in different dietary models of the same genotype.

^c^ indicates that there is a statistical difference in breast milk PUFAs between model 1 and model 2 with the same genotype.

^d^ indicates that there is a statistical difference in breast milk PUFAs between model 1 and model 3 with the same genotype.

^e^ indicates that there is a statistical difference in breast milk PUFAs between model 2 and model 3 with the same genotype.

**Supplementary Table 5 Effects of rs1535 dominant pattern of *FADS2* gene and different dietary patterns on fatty acid concentration in breast milk**

| *FADS2*  rs1535 | LA | GLA | ALA | DGLA | AA | EPA | DTA | DHA |
| --- | --- | --- | --- | --- | --- | --- | --- | --- |
| AA+M1 | 0.373±0.208 | 0.043  (0.023,0.066) | 0.136±0.074 | 0.055±0.030 | 0.078  (0.047,0.114) | 0.008  (0.005,0.013) | 0.018±0.010 | 0.049±0.028 |
| AGGG+M1 | 0.356±0.192 | 0.037  (0.022,0.051) | 0.148±0.088 | 0.046±0.025 | 0.068  (0.045,0.097) | 0.008  (0.005,0.010) | 0.016±0.008 | 0.045±0.021 |
| AA+M2 | 0.389±0.223 | 0.042±0.025 | 0.151±0.089 | 0.053  (0.030,0.079) | 0.092±0.054 | 0.007  (0.003,0.012) | 0.021  (0.010,0.028) | 0.053±0.031 |
| AGGG+M2 | 0.313±0.135 | 0.033±0.016^a^ | 0.126±0.062 | 0.040  (0.026,0.062) | 0.061±0.028^a^ | 0.006  (0.004,0.010) | 0.014  (0.010,0.021)^a^ | 0.041±0.020 |
| AA+M3 | 0.390±0.185 | 0.042  (0.029,0.063) | 0.148±0.072 | 0.059  (0.041,0.081) | 0.093±0.041 | 0.009  (0.006,0.013) | 0.021±0.009 | 0.058  (0.036,0.074) |
| AGGG+M3 | 0.415±0.190^bde^ | 0.039  (0.024,0.049) | 0.159±0.069 | 0.054  (0.037,0.070)^bde^ | 0.083±0.039^be^ | 0.007  (0.005,0.011) | 0.020±0.010^bde^ | 0.051  (0.035,0.070)^be^ |

^a^ indicates that there is a statistical difference in the content of breast milk PUFAs in different genotypes of the same dietary model.

^b^ indicates that there is a statistical difference in breast milk PUFAs in different dietary models of the same genotype.

^c^ indicates that there is a statistical difference in breast milk PUFAs between model 1 and model 2 with the same genotype.

^d^ indicates that there is a statistical difference in breast milk PUFAs between model 1 and model 3 with the same genotype.

^e^ indicates that there is a statistical difference in breast milk PUFAs between model 2 and model 3 with the same genotype.

**Supplementary Table 6 Effects of rs174575 dominant pattern of *FADS2* gene and different dietary patterns on fatty acid concentration in breast milk**

| *FADS2*  rs174575 | LA | GLA | ALA | DGLA | AA | EPA | DTA | DHA |
| --- | --- | --- | --- | --- | --- | --- | --- | --- |
| CC+M1 | 0.352±0.201 | 0.043±0.026 | 0.134±0.078 | 0.050±0.029 | 0.075±0.041 | 0.008  (0.005,0.011) | 0.017±0.010 | 0.047±0.026 |
| CGGG+M1 | 0.441±0.188 | 0.041±0.023 | 0.191±0.089^a^ | 0.051±0.027 | 0.079±0.032 | 0.008  (0.006,0.010) | 0.018±0.007 | 0.050±0.020 |
| CC+M2 | 0.349±0.190 | 0.037±0.022 | 0.136±0.077 | 0.051±0.031 | 0.077±0.046 | 0.006  (0.003,0.010) | 0.017  (0.010,0.024) | 0.040  (0.025,0.063) |
| CGGG+M2 | 0.344±0.124 | 0.038±0.016 | 0.146±0.061 | 0.050±0.021 | 0.065±0.030 | 0.007  (0.006,0.011) | 0.019  (0.011,0.021) | 0.051  (0.028,0.061) |
| CC+M3 | 0.397±0.190 | 0.040  (0.026,0.059) | 0.150±0.072 | 0.063±0.031^bde^ | 0.091±0.041^bde^ | 0.008  (0.006,0.013)^be^ | 0.022±0.010^abde^ | 0.055  (0.035,0.075)^bde^ |
| CGGG+M3 | 0.445±0.189 | 0.043  (0.026,0.056) | 0.175±0.071 | 0.054±0.027 | 0.081±0.035 | 0.008  (0.005,0.014) | 0.018±0.007 | 0.054  (0.040,0.066) |

^a^ indicates that there is a statistical difference in the content of breast milk PUFAs in different genotypes of the same dietary model.

^b^ indicates that there is a statistical difference in breast milk PUFAs in different dietary models of the same genotype.

^c^ indicates that there is a statistical difference in breast milk PUFAs between model 1 and model 2 with the same genotype.

^d^ indicates that there is a statistical difference in breast milk PUFAs between model 1 and model 3 with the same genotype.

^e^ indicates that there is a statistical difference in breast milk PUFAs between model 2 and model 3 with the same genotype.

**Supplementary Table 7 Effects of rs174602 dominant pattern of *FADS2* gene and different dietary patterns on fatty acid concentration in breast milk**

| *FADS2*  rs174602 | LA | GLA | ALA | DGLA | AA | EPA | DTA | DHA |
| --- | --- | --- | --- | --- | --- | --- | --- | --- |
| TT+M1 | 0.366±0.196 | 0.044±0.026 | 0.139±0.081 | 0.054±0.030 | 0.079±0.043 | 0.008  (0.005,0.014) | 0.018±0.010 | 0.047±0.025 |
| TCCC+M1 | 0.370±0.204 | 0.040±0.026 | 0.146±0.076 | 0.046±0.025 | 0.071±0.033 | 0.007  (0.005,0.010) | 0.017±0.008 | 0.048±0.027 |
| TT+M2 | 0.371±0.195 | 0.039±0.022 | 0.143±0.079 | 0.053±0.032 | 0.082±0.047 | 0.006  (0.004,0.010) | 0.019  (0.010,0.026) | 0.048±0.027 |
| TCCC+M2 | 0.302±0.144 | 0.032±0.017 | 0.125±0.068 | 0.046±0.024 | 0.062±0.024^a^ | 0.006  (0.003,0.010) | 0.017  (0.009,0.021) | 0.043±0.024 |
| TT+M3 | 0.402±0.179 | 0.042  (0.029,0.061) | 0.141  (0.099,0.191) | 0.058  (0.041,0.082) | 0.094±0.041 | 0.009  (0.006,0.014)^be^ | 0.022±0.009^bd^ | 0.057  (0.038,0.072)^bde^ |
| TCCC+M3 | 0.413±0.206^be^ | 0.039  (0.025,0.049) | 0.143  (0.120,0.208) | 0.053  (0.033,0.069) | 0.081±0.039^a^ | 0.007  (0.005,0.011) | 0.020±0.011 | 0.047  (0.034,0.070) |

^a^ indicates that there is a statistical difference in the content of breast milk PUFAs in different genotypes of the same dietary model.

^b^ indicates that there is a statistical difference in breast milk PUFAs in different dietary models of the same genotype.

^c^ indicates that there is a statistical difference in breast milk PUFAs between model 1 and model 2 with the same genotype.

^d^ indicates that there is a statistical difference in breast milk PUFAs between model 1 and model 3 with the same genotype.

^e^ indicates that there is a statistical difference in breast milk PUFAs between model 2 and model 3 with the same genotype.

**Supplementary Table 8 Effects of rs3834458 dominant pattern of *FADS2* gene and different dietary patterns on fatty acid concentration in breast milk**

| *FADS2*  rs3834458 | LA | GLA | ALA | DGLA | AA | EPA | DTA | DHA |
| --- | --- | --- | --- | --- | --- | --- | --- | --- |
| TT+M1 | 0.372±0.205 | 0.043  (0.024,0.064) | 0.136±0.073 | 0.055±0.030 | 0.081±0.043 | 0.008(0.005,0.013) | 0.018±0.010 | 0.049±0.028 |
| T/DEL+M1 | 0.361±0.195 | 0.037  (0.022,0.054) | 0.149±0.089 | 0.046±0.026 | 0.070±0.034 | 0.008  (0.005,0.010) | 0.016±0.008 | 0.045±0.022 |
| TT+M2 | 0.399±0.218 | 0.038  (0.026,0.056) | 0.155±0.087 | 0.057±0.033 | 0.094±0.053 | 0.007  (0.004,0.012) | 0.022  (0.012,0.028) | 0.055±0.030 |
| T/DEL+M2 | 0.308±0.141^a^ | 0.030  (0.018,0.048)^a^ | 0.123±0.063 | 0.046±0.026 | 0.061±0.029^a^ | 0.006  (0.004,0.010) | 0.014  (0.010,0.021)^a^ | 0.040±0.020^a^ |
| TT+M3 | 0.395±0.185 | 0.044  (0.029,0.063) | 0.152±0.075 | 0.059  (0.041,0.081) | 0.093±0.040 | 0.009  (0.006,0.013) | 0.021±0.009 | 0.057  (0.036,0.072) |
| T/DEL+M3 | 0.418±0.195^be^ | 0.037  (0.023,0.051) | 0.158±0.071^be^ | 0.054  (0.036,0.069) | 0.084±0.040^bde^ | 0.007  (0.005,0.011) | 0.020±0.010^bde^ | 0.053  (0.035,0.073)^be^ |

^a^ indicates that there is a statistical difference in the content of breast milk PUFAs in different genotypes of the same dietary model.

^b^ indicates that there is a statistical difference in breast milk PUFAs in different dietary models of the same genotype.

^c^ indicates that there is a statistical difference in breast milk PUFAs between model 1 and model 2 with the same genotype.

^d^ indicates that there is a statistical difference in breast milk PUFAs between model 1 and model 3 with the same genotype.

^e^ indicates that there is a statistical difference in breast milk PUFAs between model 2 and model 3 with the same genotype.

**Supplementary Table 9 Effects of rs498793 dominant pattern of *FADS2* gene and different dietary patterns on fatty acid concentration in breast milk**

| *FADS2*  rs498793 | LA | GLA | ALA | DGLA | AA | EPA | DTA | DHA |
| --- | --- | --- | --- | --- | --- | --- | --- | --- |
| TT+M1 | 0.368±0.194 | 0.043±0.026 | 0.143±0.081 | 0.051±0.028 | 0.076±0.039 | 0.008  (0.005,0.011) | 0.017±0.009 | 0.047±0.024 |
| TCCC+M1 | 0.316±0.285 | 0.038±0.025 | 0.125±0.093 | 0.043±0.032 | 0.069±0.048 | 0.009  (0.002,0.011) | 0.014±0.009 | 0.053±0.036 |
| TT+M2 | 0.354±0.189 | 0.038±0.022 | 0.138±0.077 | 0.051±0.032 | 0.074  (0.044,0.102) | 0.006  (0.003,0.010) | 0.017  (0.010,0.024) | 0.047±0.027 |
| TCCC+M2 | 0.312±0.150 | 0.033±0.017 | 0.130±0.069 | 0.048±0.022 | 0.068  (0.034,0.083) | 0.007  (0.003,0.010) | 0.018  (0.009,0.020) | 0.044±0.021 |
| TT+M3 | 0.393±0.184 | 0.039  (0.026,0.056) | 0.150±0.069 | 0.054  (0.038,0.077)^bde^ | 0.086±0.040 | 0.008  (0.005,0.013)^bce^ | 0.021±0.010^bd^ | 0.053  (0.035,0.068)^bde^ |
| TCCC+M3 | 0.466±0.196^be^ | 0.042  (0.026,0.074) | 0.178±0.075 | 0.060  (0.038,0.070) | 0.099±0.041^be^ | 0.009  (0.006,0.012) | 0.022±0.010^bde^ | 0.061  (0.042,0.084) |

^a^ indicates that there is a statistical difference in the content of breast milk PUFAs in different genotypes of the same dietary model.

^b^ indicates that there is a statistical difference in breast milk PUFAs in different dietary models of the same genotype.

^c^ indicates that there is a statistical difference in breast milk PUFAs between model 1 and model 2 with the same genotype.

^d^ indicates that there is a statistical difference in breast milk PUFAs between model 1 and model 3 with the same genotype.

^e^ indicates that there is a statistical difference in breast milk PUFAs between model 2 and model 3 with the same genotype.

**Supplementary Table 10 Effects of rs1000778 dominant pattern of *FADS3* gene and different dietary patterns on fatty acid concentration in breast milk**

| *FADS3*  rs1000778 | LA | GLA | ALA | DGLA | AA | EPA | DTA | DHA |
| --- | --- | --- | --- | --- | --- | --- | --- | --- |
| GG+M1 | 0.350±0.208 | 0.042±0.028 | 0.135±0.084 | 0.049  (0.025,0.071) | 0.074±0.041 | 0.008  (0.004,0.011) | 0.017±0.010 | 0.046±0.027 |
| GAAA+M1 | 0.388±0.187 | 0.042±0.021 | 0.151±0.071 | 0.050  (0.034,0.066) | 0.078±0.037 | 0.008  (0.006,0.011) | 0.017±0.009 | 0.049±0.023 |
| GG+M2 | 0.354±0.207 | 0.039±0.023 | 0.138±0.085 | 0.052±0.031 | 0.079±0.048 | 0.006  (0.003,0.011) | 0.019±0.012 | 0.040  (0.023,0.063) |
| GAAA+M2 | 0.335±0.118 | 0.033±0.016 | 0.136±0.055 | 0.049±0.026 | 0.067±0.030 | 0.007  (0.003,0.010) | 0.017±0.007 | 0.048  (0.028,0.064) |
| GG+M3 | 0.375±0.166 | 0.039  (0.027,0.056) | 0.140  (0.105,0.187) | 0.054  (0.038,0.080) | 0.088±0.039 | 0.008  (0.005,0.013) | 0.021±0.010^bd^ | 0.051  (0.036,0.071)^bde^ |
| GAAA+M3 | 0.451±0.214^abe^ | 0.041  (0.023,0.059) | 0.148  (0.122,0.235) | 0.058  (0.038,0.072) | 0.087±0.040 | 0.008  (0.005,0.012) | 0.020±0.009 | 0.056  (0.034,0.073) |

^a^ indicates that there is a statistical difference in the content of breast milk PUFAs in different genotypes of the same dietary model.

^b^ indicates that there is a statistical difference in breast milk PUFAs in different dietary models of the same genotype.

^c^ indicates that there is a statistical difference in breast milk PUFAs between model 1 and model 2 with the same genotype.

^d^ indicates that there is a statistical difference in breast milk PUFAs between model 1 and model 3 with the same genotype.

^e^ indicates that there is a statistical difference in breast milk PUFAs between model 2 and model 3 with the same genotype.

**Supplementary Table 11 Effects of rs174450 dominant pattern of *FADS3* gene and different dietary patterns on fatty acid concentration in breast milk**

| *FADS3*  rs174450 | LA | GLA | ALA | DGLA | AA | EPA | DTA | DHA |
| --- | --- | --- | --- | --- | --- | --- | --- | --- |
| AA+M1 | 0.362±0.202 | 0.043±0.026 | 0.138±0.083 | 0.052±0.029 | 0.074  (0.043,0.107) | 0.008  (0.005,0.011) | 0.018±0.010 | 0.048±0.027 |
| AGGG+M1 | 0.369±0.199 | 0.041±0.026 | 0.146±0.080 | 0.049±0.028 | 0.0.74  (0.048,0.098) | 0.008  (0.005,0.011) | 0.016±0.009 | 0.046±0.021 |
| AA+M2 | 0.357±0.208 | 0.038  (0.022,0.052) | 0.140±0.085 | 0.053  (0.026,0.072) | 0.080±0.049 | 0.006  (0.004,0.011) | 0.019  (0.009,0.026) | 0.047±0.028 |
| AGGG+M2 | 0.331±0.128 | 0.029  (0.019,0.044) | 0.133±0.059 | 0.041  (0.033,0.061) | 0.065±0.031 | 0.006  (0.005,0.010) | 0.017  (0.011,0.021) | 0.045±0.023 |
| AA+M3 | 0.354  (0.255,0.532) | 0.040  (0.026,0.059) | 0.148±0.065 | 0.056  (0.039,0.081) | 0.090±0.039 | 0.009  (0.006,0.014) | 0.021±0.010 | 0.057  (0.036,0.076)^bde^ |
| AGGG+M3 | 0.385  (0.315,0.530)^be^ | 0.040  (0.028,0.049) | 0.162±0.075 | 0.054  (0.038,0.073) | 0.086±0.040^be^ | 0.007  (0.005,0.011) | 0.020±0.009^bd^ | 0.053  (0.035,0.067) |

^a^ indicates that there is a statistical difference in the content of breast milk PUFAs in different genotypes of the same dietary model.

^b^ indicates that there is a statistical difference in breast milk PUFAs in different dietary models of the same genotype.

^c^ indicates that there is a statistical difference in breast milk PUFAs between model 1 and model 2 with the same genotype.

^d^ indicates that there is a statistical difference in breast milk PUFAs between model 1 and model 3 with the same genotype.

^e^ indicates that there is a statistical difference in breast milk PUFAs between model 2 and model 3 with the same genotype.

**Supplementary Table 12 Effects of rs7115739 dominant pattern of *FADS3* gene and different dietary patterns on fatty acid concentration in breast milk**

| *FADS3*  rs7115739 | LA | GLA | ALA | DGLA | AA | EPA | DTA | DHA |
| --- | --- | --- | --- | --- | --- | --- | --- | --- |
| GG+M1 | 0.365±0.202 | 0.043±0.026 | 0.140±0.083 | 0.051±0.029 | 0.078±0.042 | 0.008  (0.005,0.011) | 0.017±0.010 | 0.048±0.027 |
| GTTT+M1 | 0.363±0.199 | 0.041±0.025 | 0.145±0.079 | 0.050±0.029 | 0.070±0.033 | 0.008  (0.005,0.011) | 0.016±0.008 | 0.046±0.021 |
| GG+M2 | 0.349±0.206 | 0.039±0.023 | 0.137±0.084 | 0.050±0.031 | 0.077±0.048 | 0.006  (0.004,0.011) | 0.018±0.012 | 0.040  (0.023,0.061) |
| GTTT+M2 | 0.343±0.122 | 0.033±0.017 | 0.135±0.057 | 0.052±0.027 | 0.070±0.031 | 0.007  (0.004,0.010) | 0.017±0.007 | 0.048  (0.028,0.068) |
| GG+M3 | 0.395±0.185 | 0.042  (0.026,0.060) | 0.148  (0.107,0.191) | 0.056  (0.038,0.081)^bde^ | 0.089±0.040 | 0.008  (0.006,0.013) | 0.021±0.010^bd^ | 0.055  (0.036,0.071)^bde^ |
| GTTT+M3 | 0.427±0.198 | 0.037  (0.026,0.049) | 0.146  (0.119,0.234) | 0.055  (0.036,0.073) | 0.085±0.041 | 0.007  (0.005,0.014) | 0.019±0.009 | 0.053  (0.035,0.075) |

^a^ indicates that there is a statistical difference in the content of breast milk PUFAs in different genotypes of the same dietary model.

^b^ indicates that there is a statistical difference in breast milk PUFAs in different dietary models of the same genotype.

^c^ indicates that there is a statistical difference in breast milk PUFAs between model 1 and model 2 with the same genotype.

^d^ indicates that there is a statistical difference in breast milk PUFAs between model 1 and model 3 with the same genotype.

^e^ indicates that there is a statistical difference in breast milk PUFAs between model 2 and model 3 with the same genotype.
